# Oral herbal medicine for breast cancer-related symptoms: an evidence map of systematic reviews

**DOI:** 10.3389/fmed.2026.1903933

**Published:** 2026-08-05

**Authors:** Ji Hee Jun, Tae-Young Choi, Changsop Yang, Hye Won Lee, Myeong Soo Lee

**Affiliations:** 1 KM Science Research Division, Korea Institute of Oriental Medicine, Daejeon, Republic of Korea; 2 KM Convergence Research Division, Korea Institute of Oriental Medicine, Daejeon, Republic of Korea

**Keywords:** breast cancer, bubble chart, evidence map, evidence synthesis, herbal medicine, systematic review

## Abstract

**Background:**

Breast cancer remains the most commonly diagnosed malignancy in women worldwide. While the use of herbal medicine as a complementary approach has expanded, the existing literature is highly heterogeneous, and a comprehensive overview mapping the research landscape is lacking.

**Objective:**

This evidence map identifies the evidence and research gaps in herbal medicine for breast cancer.

**Methods:**

Ten electronic databases were systematically searched from inception to 30 April 2025. Systematic reviews (SRs) and meta-analyses of randomized controlled trials (RCTs) evaluating herbal medicine for breast cancer patients were included. Two reviewers independently screened the literature, selected eligible studies, and extracted data, and the methodological quality of the included reviews was assessed using AMSTAR 2. The included SRs were stratified into two groups based on the timing of herbal medicine administration (“Undergoing treatment” and “Mixed / Both phase”) and visualized as a bubble chart using custom HTML5 and Scalable Vector Graphics (SVG) code, developed with the assistance of Claude (Claude sonnet 4.6, Anthropic). In this map, the treatment phase and clinical condition are plotted on the *x*-axis and 12 predefined outcome domains on the *y*-axis, with bubble color indicating the direction of effect, and bubble size representing the number of SRs.

**Results:**

A total of 31 SRs were included, published between 2012 and 2025. The included reviews were more concentrated in the “Undergoing treatment” phase (*n* = 27) than in the “Mixed / Both phase” (*n* = 4). Most SRs addressed non-symptom-specific chemotherapy outcomes (*n* = 13), followed by menopausal-like symptoms (*n* = 4), with predominantly positive findings. According to AMSTAR 2, 28 SRs (90.3%) were rated as “Critically Low” quality and 3 SRs (9.7%) as “Low” quality, with critical deficits in protocol registration and risk of bias consideration.

**Conclusion:**

This evidence map provides the first comprehensive visual overview of SRs on herbal medicine for breast cancer. While clustering around treatment-related symptom management, the methodological quality is consistently low. Future research must prioritize high-quality trials in under-investigated domains and long-term, patient-centered outcomes, using standardized protocols and rigorous adherence to international reporting guidelines.

**Systematic review registration:**

https://www.crd.york.ac.uk/prospero/display_record.php?ID=CRD420261330650, identifier CRD420261330650.

## Introduction

Breast cancer is the most frequently diagnosed malignancy among women worldwide and remains a leading cause of cancer-related mortality (1, 2). In Korea, its incidence has continued to rise, driven by multiple risk factors, including the westernization of dietary patterns, declining fertility rates, delayed menarche and menopause, obesity, and increased exposure to endogenous and exogenous hormones (3, 4).

The standard treatment for breast cancer includes surgery, chemotherapy, radiotherapy, endocrine therapy, and targeted therapy. Advances in early detection and therapeutic strategies have substantially improved survival outcomes (5, 6). However, chemotherapeutic agents, while selectively destructive to malignant tissues also damage healthy tissue, resulting in adverse effects that negatively impact patients’ treatment compliance (7). In response, an increasing number of breast cancer patients have turned to complementary and integrative approaches alongside conventional treatment (8, 9).

Among these complementary therapies, herbal medicine, particularly traditional East Asian medicine, is one of the most frequently used modalities (10, 11). Herbal medicine aims to control toxicities and side effects of cancer treatment and improve patients’ quality of life, and has been reported to play potential role in enhancing immune function, reducing chemotherapy-related adverse effects, and prolonging survival in breast cancer patients (9, 12, 13). Beyond herbal medicine, other natural bioactive compounds have also been investigated for their potential role in breast cancer prevention and management. For instance, polyphenols—abundant in the Mediterranean diet—have been associated with antioxidant, anti-inflammatory, and anti-tumorigenic properties relevant to cancer care (14).

An evidence map is a structured approach to evidence synthesis that systematically identifies, organizes, and visualizes the existing body of research within a defined topic area (15). Unlike conventional systematic reviews (SRs) or meta-analyses, which focus on quantitatively synthesizing effect sizes for specific interventions, mapping reviews and evidence and gap maps address broad research questions and aim to describe the overall landscape rather than answer specific questions about intervention effectiveness (16).

Although research on herbal medicine for breast cancer has been steadily increasing, the existing studies are highly heterogeneous in terms of populations, interventions, comparators, and outcome measures, making it difficult to grasp the overall landscape of the available evidence and to distinguish between well-established and under-investigated areas (9). Moreover, to date, no comprehensive evidence map has been conducted on herbal medicine for breast cancer.

Therefore, this study aims to systematically identify, categorize, and visualize the existing research on herbal medicine for breast cancer as a bubble chart, in order to clarify the distribution of evidence, highlight methodological limitations, and identify priority areas for future research.

## Methods

### Eligibility criteria

Eligibility criteria were predefined using the PICO (Population, Intervention, Comparator, and Outcome) framework. We included systematic reviews and meta-analyses of randomized controlled trials evaluating herbal medicine for breast cancer patients at any stage. Interventions of interest were oral herbal medicines, including single-herb extracts, multi-herb decoctions or granules, and standardized patent herbal medicines (Zhōngchéngyào), used either as monotherapy or as an adjuvant to conventional cancer treatments. Reviews in which the primary studies predominantly involved non-oral routes of administration, such as topical/external preparations or intravenous injections, were excluded. Comparators included placebo, conventional therapy alone, or no treatment. To be eligible, studies had to report at least one clinical outcome, such as total effective rate, quality of life, or adverse events. Study protocols, conference abstracts, and primary clinical trials were excluded. The protocol for this evidence map was prospectively registered in PROSPERO (CRD420261330650).

### Literature search

Ten electronic databases were searched from inception to 30 April 2025: PubMed, Embase, Cochrane Library, Allied and Complementary Medicine Database (AMED), China National Knowledge Infrastructure (CNKI), Wanfang, VIP, Research Information Service System (RISS), Oriental Search Integrated System (OASIS), and Korean Studies Information Services System (KISS). All database searches were rerun and updated before data synthesis to ensure inclusion of the most recent publications. Search strategies combined keywords, subject headings, and index terms related to herbal medicine and breast cancer–related symptoms. The detailed search strategy is presented in Supplementary Material 1.

### Study selection and data extraction

Two reviewers (JJ and T-YC) independently screened the titles and abstracts of all identified records against the predefined inclusion and exclusion criteria. The full texts of potentially eligible studies were then retrieved and independently assessed by the same two reviewers for final inclusion. Any disagreements between the reviewers were resolved through discussion, and a third reviewer was consulted when consensus could not be reached (17).

### Quality assessment

The methodological quality of the included systematic reviews was independently assessed by two reviewers using the AMSTAR 2 (A MeaSurement Tool to Assess Systematic Reviews 2) tool (18). AMSTAR 2 consists of 16 items, including seven critical domains (Items 2, 4, 7, 9, 11, 13, and 15) that significantly impact the overall validity of a review. Based on the assessment of these critical and non-critical items, the overall confidence in the results of each review was categorized into four levels: “High,” “Moderate,” “Low,” or “Critically Low.” Any discrepancies between the reviewers during the assessment process were resolved through discussion or by consulting a third reviewer.

### Data synthesis

Data synthesis was conducted by categorizing the included studies according to study characteristics, including population, intervention type, comparator, and outcome measures. To examine the distribution of evidence across different clinical contexts, the studies were further stratified into two groups based on the timing of herbal medicine administration: (1) “Undergoing Treatment,” referring to herbal medicine administered concurrently with active conventional treatment (e.g., chemotherapy, radiotherapy, endocrine therapy, or surgery); and (2) “Mixed/Both Phase,” referring to SRs in which the included primary RCTs spanned both the active-treatment and post-treatment periods (i.e., interventions were heterogeneous across primary studies with respect to treatment timing, or continued from the active treatment period into the post-treatment period).

Data extracted from included systematic reviews were compiled in Microsoft Excel and subsequently visualized as an evidence map. The map was constructed as a bubble chart in which the *x*-axis represents clinical conditions stratified by treatment phase (Undergoing treatment vs. Mixed/Both phase), the *y*-axis represents predefined outcome domains (Domains 1–12, detailed in Table 1). The direction of effect for each cell was assigned based on the majority of outcomes reported within that domain. When an SR reported mixed or multiple outcomes within a single outcome domain, two reviewers independently determined the predominant direction of effect (blue: positive; orange: inconclusive; red: no effect), and any discrepancies were resolved through discussion. Bubble size reflects the number of SRs contributing to each cell (i.e., each treatment phase and clinical condition × outcome domian combination), not the number of primary RCTs included within those reviews. The figure was produced using HTML5, Scalable Vector Graphics (SVG), and JavaScript, with coding assistance from Claude (Claude sonnet 4.6, Anthropic). Data processing was performed in Python 3 (Python Software Foundation).

**TABLE 1:** Twelve outcome domains used in the evidence map.

Domain no.	Outcome domain
1	Efficacy
2	Pain
3	Function
4	Quality of life
5	Prognosis (Survival and recurrence)
6	Immune function
7	Endocrine-related outcomes
8	Safety-gastrointestinal
9	Safety-hematologic and metabolic
10	Safety-organ toxicity
11	Safety-dermatologic
12	Safety-general

To assess the degree of overlap among SRs addressing similar research questions, we constructed a citation matrix and calculated the Corrected Covered Area (CCA) for the cluster of SRs classified under “non-symptom specific (general chemotherapy)” (*n* = 13), as this domain showed the highest concentration of reviews and the greatest *a priori* likelihood of overlapping primary studies. The CCA was calculated using the following formula: CCA = (N − r)/(r × c − r). where N is the total number of cells coded 1 across the matrix, r is the number of unique primary RCTs (rows), and c is the number of SRs (columns). CCA values were interpreted according to established thresholds: 0%–5% slight, 6%–10% moderate, 11%–15% high, and >15% very high overlap (19).

## Results

### Study screening

**FIGURE 1:**
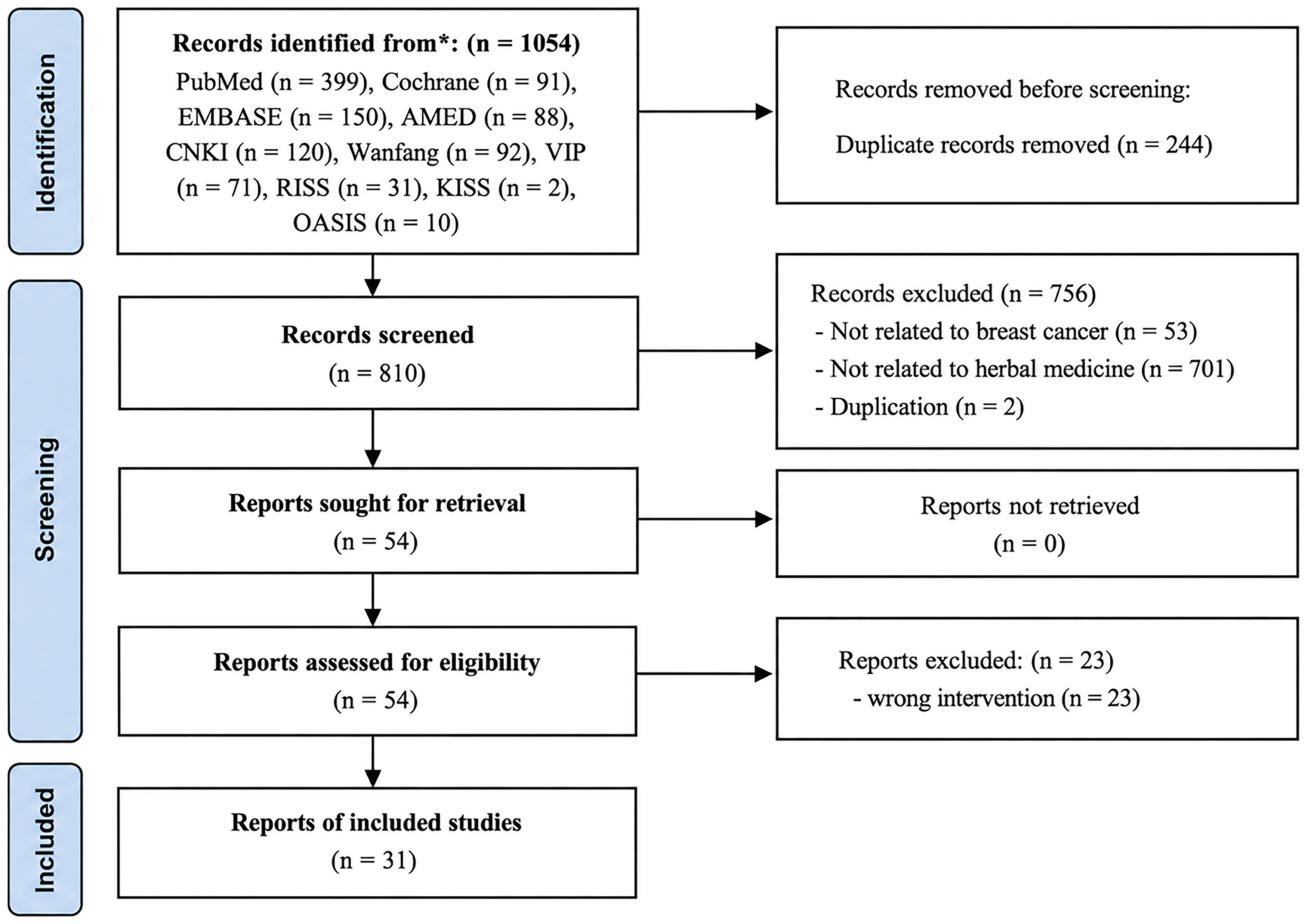
Flow chart of trial selection process.

The literature search identified 1,054 records. After removing 244 duplicates, 810 titles and abstracts were screened, and 756 articles were excluded for not meeting the eligibility criteria. Fifty-four full-text articles were then assessed for eligibility, and 23 articles were further excluded with reasons provided. Ultimately, 31 SRs (20–50) met the inclusion criteria and were included in this evidence map. The detailed study selection process is shown in the PRISMA flow diagram (Figure 1).

### Characteristics of included reviews

The 31 (20–50) included SRs were published between 2012 and 2025 and originated from three countries (China, South Korea, and Australia), with the most conducted in China (20–28, 30–36, 38–50). The reviews evaluated a wide range of herbal medicine interventions, including traditional herbal decoctions and single-herb extracts, most of which were administered as adjunctive therapy alongside conventional treatments such as chemotherapy, endocrine therapy, or radiotherapy. The included systematic reviews covered a broad range of breast cancer-related conditions, with interventions predominantly consisting of herbal medicine administered concurrently with conventional treatments such as chemotherapy, radiotherapy, and endocrine therapy. Detailed characteristics of the included reviews, including interventions, comparators, number of included trials, sample sizes, and overall methodological confidence ratings, are summarized in Table 2.

**TABLE 2:** General characteristics of the included systematic reviews.

References	Country	Breast cancer-related condition	Intervention	Comparator	Included trials (*n*)	Sample size (*n*)	Overall confidence**
Undergoing treatment (n = 27)							
Xue et al. (20)	China	Anthracycline-induced cardiotoxicity	HM + (C)	Chemotherapy	10 RCTs	648	Critically Low
Yang et al. (21)	China	Bone metastasis	HM	WM	14 RCTs	881	Critically Low
Sun et al. (22)	China	Bone loss / osteoporosis	HM HM + (C)	No treatment WM	22 RCTs	1570	Critically Low
Qian et al. (23)	China	Bone metastasis	HM + (C)	Chemotherapy Radiotherapy non-antitumor therapy	13 RCTs	879	Critically Low
Pan et al. (24)	China	Chemotherapy-related adverse effects	Modified xiaoyao san + (C)	Chemotherapy	17 RCTs	1207	Low
Liu et al. (25)	China	Chemotherapy-induced cardiotoxicity	HM + (C)	Chemotherapy	42 RCTs	2847	Critically Low
Quan et al. (26)	China	Endocrine therapy-induced osteoporosis/bone loss	HM + (C)	Adjuvant endocrine therapy	38 RCT	2170	Critically Low
Dong et al. (27)	China	HER-2 + Targeted therapy	HM + (C)	Chemotherapy	12 RCTs	1140	Critically Low
Fang et al. (28)	China	HER-2 + Targeted therapy	HM + (C)	WM	12 RCTs	1057	Critically Low
Li et al. (29)	China, Australia	Hot flushes induced by endocrine therapy	HM + Endocrine therapy	Placebo No treatment HM	5 RCTs	397	Critically Low
Shao et al. (30)	China	Menopausal-like syndrome	HM + (C)	Endocrine therapy	21 RCTs	1617	Critically Low
Lin et al. (31)	China	Menopausal-like syndrome	HM + (C)	Placebo WM	25 RCTs	1485	Critically Low
Mao et al. (32)	China	TNBC (IV stage)	HM + (C)	Chemotherapy	22 RCTs	1739	Critically Low
Zhu et al. (33)	China	TNBC (mixed stage)	HM + (C)	Chemotherapy	22 RCTs	1540	Critically Low
Lin et al. (34)	China	TNBC (mixed stage)	Fuzheng xiaoliu decoction + (C)	Chemotherapy	14 RCTs	1179	Critically Low
Li et al. (35)	China	Not symptom-specific (general chemotherapy)	HM	n.r.	8 RCTs	221	Critically Low
Cheng et al. (36)	China	Not symptom-specific (general chemotherapy)	HM + (C)	Chemotherapy	13 RCTs	1053	Critically Low
Kim et al. (37)	Korea	Not symptom-specific (general chemotherapy)	HM + (C)	Radiotherapy Chemotherapy	8 RCTs	798	Critically Low
Li et al. (38)	China	Not symptom-specific (general chemotherapy)	Fuzheng xiaoliu decoction + (C)	Placebo No treatment WM	15 RCTs	1258	Critically Low
Yang et al. (39)	China	Not symptom specific (general chemotherapy)	Pingxiao capsule + (C)	Adjuvant endocrine therapy Chemotherapy	15 RCTs	1272	Critically Low
Xie et al. (40)	China	Not symptom specific (general chemotherapy)	Pingxiao jiaonang + (C)	Chemotherapy Radiotherapy non-antitumor therapy	15 RCTs	1402	Critically Low
Wang et al. (41)	China	Not symptom specific (general chemotherapy)	Shenyi jiaonang + (C)	WM Chemotherapy	4 RCTs	274	Critically Low
Ma et al. (42)	China	Not symptom-specific (general chemotherapy)	Yanghe decoction + (C)	Chemotherapy	10 RCTs	691	Critically Low
Xie et al. (43)	China	Not symptom-specific (general chemotherapy)	Xiaoyao san + (C)	Chemotherapy	16 RCTs	1107	Critically Low
Li et al. (44)	China	Not symptom specific (general chemotherapy)	Xihuang pill / capsule + (C)	Adjuvant endocrine therapy	12 RCTs	1260	Critically Low
Lu et al. (45)	China	Not symptom specific (general chemotherapy)	Xihuang wan / jiaonang Xihuang wan / jiaonang + (C)	WM	10 RCTs	705	Critically Low
Mao et al. (46)	China	Not symptom specific (general chemotherapy)	Xihuang pill / capsule + (C)	Chemotherapy	13 RCTs	1272	Critically Low
Mixed / both phase (n = 4)							
Wang et al. (47)	China	Menopausal-like syndrome	HM	Placebo No treatment	42 RCTs	3112	Low
Che et al. (48)	China	Menopausal-like syndrome	HM HM + (C)	Placebo WM	13 RCTs	976	Critically Low
Wu et al. (49)	China	TNBC (I∼III stage)	HM + (C)	No treatment WM	9 RCTs	735	Critically Low
Ge et al. (50)	China	Not symptom specific (general chemotherapy)	Xihuang pill / capsule + (C)	Chemotherapy Radiotherapy non-antitumor therapy	26 RCTs	2272	Low

C, control group; HER-2, human epidermal growth factor receptor 2; HM, herbal medicine; n.r., not reported; RCT, randomized controlled trial; TNBC, Triple-negative breast cancer; WM: Western medicine **AMSTAR 2 (A MeaSurement Tool to Assess systematic Reviews) was used for quality assessment. “Critically Low” indicates more than one critical flaw in the review methodology.

### Methodological quality of SRs

**FIGURE 2:**
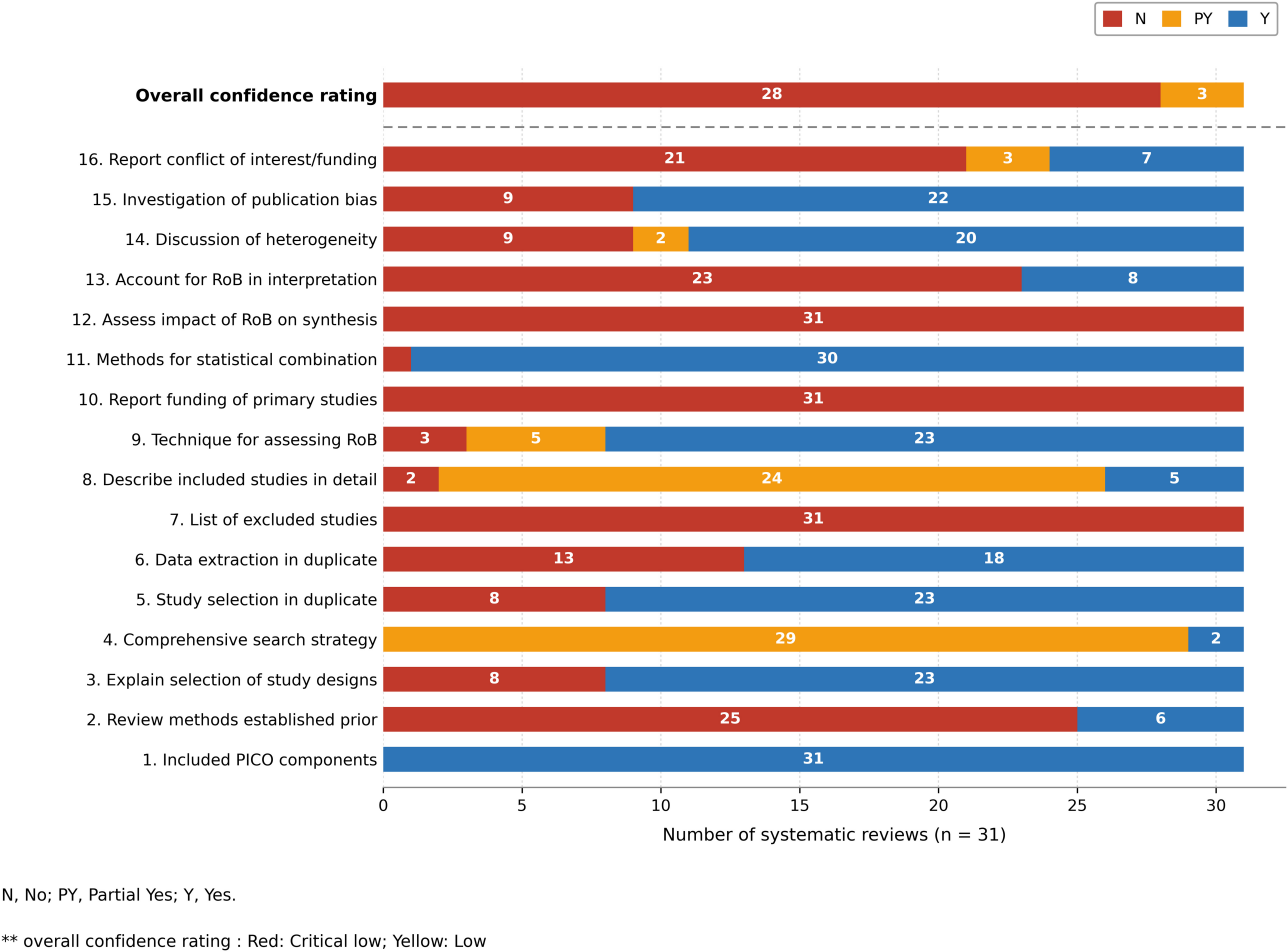
Quality assessment. AMSTAR2 was used to critically appraise the reporting quality of each included SR. The overall confidence of each SR was graded as “high” (no or non-critical weakness in all items), “moderate” (more than one non-critical weakness among all the items), “low” (one critical flaw with or without non-critical weakness), or “Critically Low” (more than one critical flaw with or without non-critical weakness).

According to AMSTAR 2, 28 SRs (90.3%) (20–23, 25–46, 48, 49) were rated as “Critically Low” quality and 3 SRs (9.7%) (24, 47, 50) as “Low” quality (Figure 2). The items with the lowest fulfillment rates were protocol registration (item 2; 25 SRs, 80.6%), providing a list of excluded studies with justifications (item 7; 31 SRs, 100%), reporting of funding sources of included primary studies (item 10; 31 SRs, 100%), assessing the impact of risk of bias on meta-analysis results (item 12; 31 SRs, 100%), and considering risk of bias when interpreting the results of individual studies (item 13; 23 SRs, 74.2%). Among these, items 7, 10, and 12 received “No” ratings from all 31 included SRs (100%). The high frequency of “Partial Yes (PY)” ratings for item 4, which concerns the comprehensiveness of the literature search strategy, was attributable to the lack of reporting on whether subject-matter experts were consulted in developing the search strategy, as well as the failure to describe the search strings used across all databases.

### Evidence maps

#### Distribution by treatment phase

**FIGURE 3:**
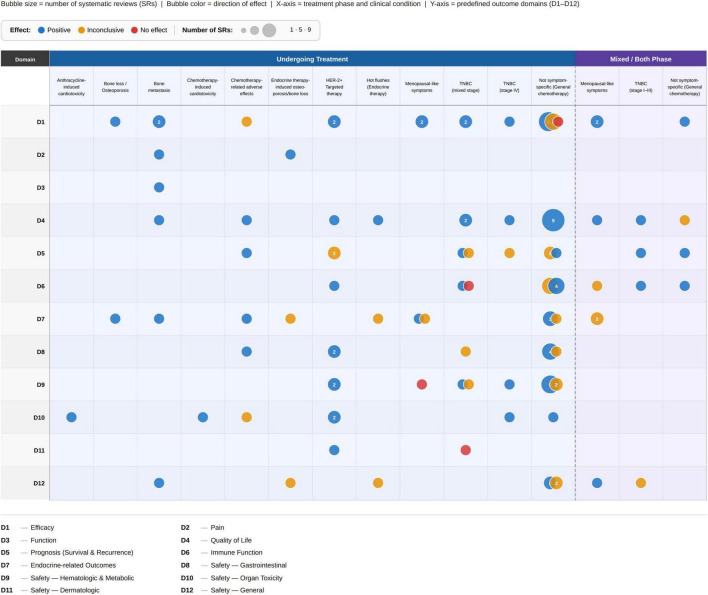
Evidence map of oral herbal medicine for breast cancer-related symptoms. D1: Efficacy; D2: Pain; D3: Function; D4: Quality of life; D5: Prognosis (Survival and Recurrence); D6: Immune function; D7: Endocrine-related outcomes; D8: Safety-Gastrointestinal; D9: Safety-Hematologic & Metabolic; D10: Safety-Organ toxicity; D11: Safety-Dermatologic; D12: Safety-General.

The included systematic reviews were categorized into two groups according to patients’ treatment status. The included studies were stratified into two groups based on the timing of herbal medicine administration: “undergoing treatment” for patients receiving active conventional treatment, and “Mixed/Both phase” for SRs in which the included primary RCTs spanned both the active-treatment and post-treatment periods. The extracted data were tabulated and visualized as an evidence map (Figure 3 and Supplementary Material 2), with clinical conditions stratified by treatment phase on the *x*-axis (horizontal axis), and outcome domains on the *y*-axis (vertical axis) and bubble color indicating the direction of effect (positive, inconclusive, or no effect).

#### Evidence distribution across clinical phases

The evidence map showed that SRs were more heavily concentrated in the “Undergoing treatment” phase (*n* = 27) (20–46) than in the “Mixed/Both phase” (*n* = 4) (47–50), indicating that research interest has predominantly focused on managing treatment-related adverse effects and toxicities during active breast cancer treatment. Within the “Undergoing treatment” phase, the condition domains encompassed a broad range of issues, including non-symptom specific conditions (general chemotherapy), menopausal-like symptoms, bone metastasis, targeted therapy or chemotherapy-related adverse effects (e.g., cardiotoxicity), and bone loss. In contrast, the “Mixed/Both phase” was limited to a narrower set of domains, specifically addressing menopausal-like symptoms, triple-negative breast cancer (TNBC), and non-symptom specific conditions.

#### Concentration of research by symptom/condition

Among all outcome domains, the largest number of SRs addressed non-symptom specific outcomes related to general chemotherapy (*n* = 13) (35–46, 50), followed by menopausal-like symptoms (*n* = 4) (30, 31, 47, 48) and bone metastasis, HER-2 + targeted therapy, and TNBC (mixed stage) (*n* = 2 each). To evaluate whether this concentration of reviews reflected a genuine accumulation of independent evidence or was partly attributable to overlapping primary studies, we performed a citation matrix analysis and calculated the Corrected Covered Area (CCA) for this cluster. Among the 13 SRs (35–44, 50) classified under non-symptom specific (general chemotherapy) outcomes, the citation matrix comprised 144 unique primary RCTs cited a total of 161 times across the included reviews, yielding a CCA of 0.98%. According to established thresholds (19), this indicates a slight degree of overlap, suggesting that the apparent concentration of evidence in this domain largely reflects independent bodies of primary research rather than substantial double-counting. In these domains, multiple SRs reported potentially positive effects of herbal medicine; however, given the generally low methodological quality of the included reviews, these findings should be interpreted with caution.

#### Areas with high volume but low methodological reliability

Although the evidence map visually demonstrates a concentration of positive findings across several domains, the methodological quality assessment using AMSTAR 2 revealed that nearly all included reviews were rated as “Low” or “Critically Low” quality. Specifically, while domains like non-symptom specific outcome (general chemotherapy) and menopausal-like symptoms are addressed by a relatively high volume of systematic reviews, these reviews frequently present with substantial methodological flaws. Consequently, despite the apparent abundance of evidence in these areas, its true reliability remains severely compromised.

#### Temporal trends in publications

**FIGURE 4:**
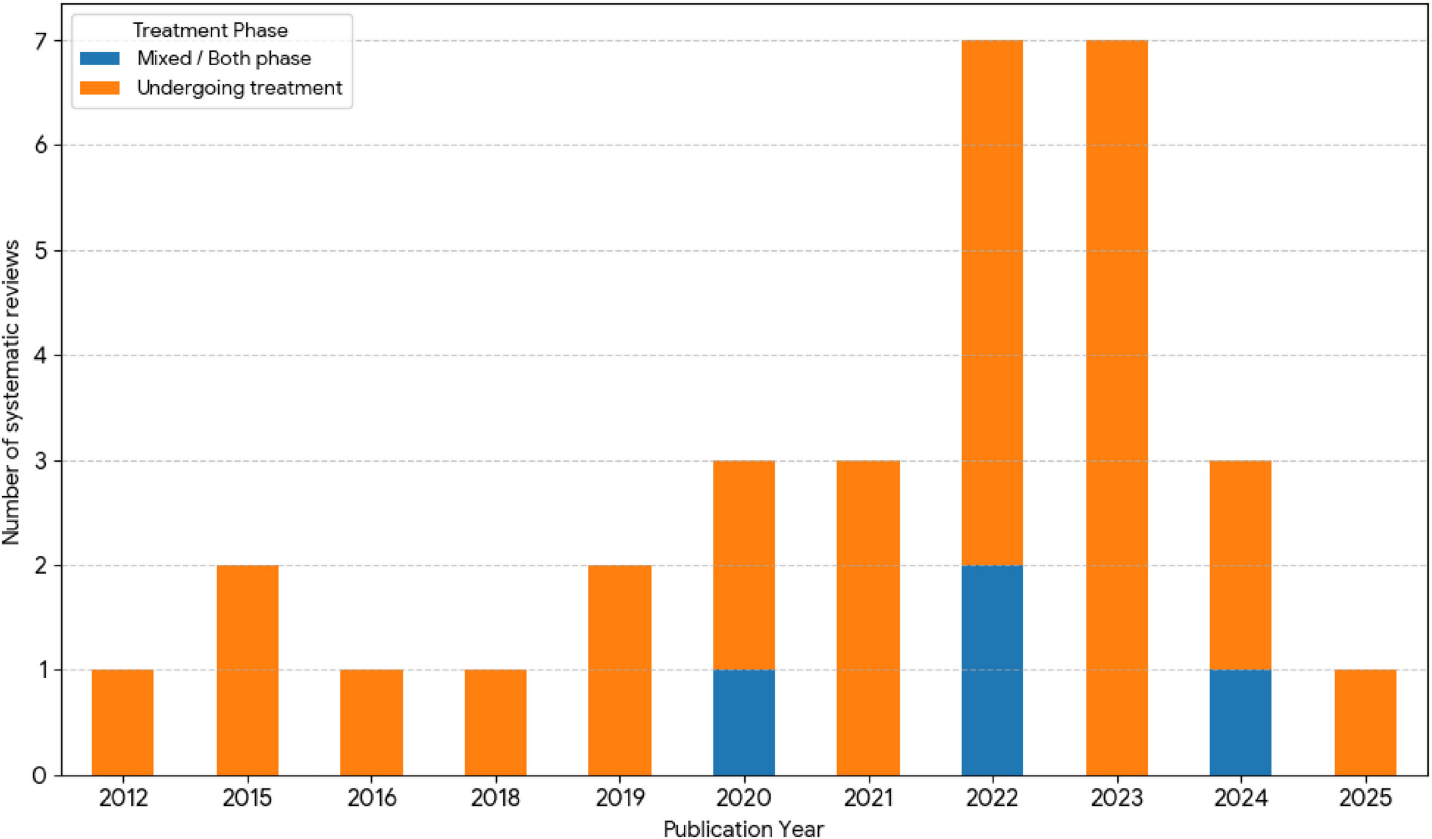
Publication year.

As shown in Figure 4, the annual number of systematic reviews on oral herbal medicine for breast cancer-related symptoms has increased substantially over time, with a notable acceleration from 2019 onward. Publications were sparse before 2019, with only one to two reviews published per year. The total annual output reached its highest level in 2022 and 2023, with seven systematic reviews published in each of these two consecutive years, followed by three reviews in 2024. This upward trend reflects a growing research interest in the role of herbal medicine as an adjunctive therapy for breast cancer patients. A category-specific analysis revealed that the “Undergoing Treatment” category accounted for the majority of reviews across all years, while the “Mixed/Both Phase” category, encompassing reviews that addressed interventions spanning both active treatment and post-treatment periods, has emerged more prominently in recent years. This pattern suggests a progressive broadening of research scope from intra-treatment management toward a more comprehensive understanding of herbal medicine use across the breast cancer treatment continuum.

## Discussion

### Overview of findings

To our knowledge, this is the first evidence map to systematically identify, categorize, and visualize the existing body of systematic reviews on herbal medicine for breast cancer. A total of 31 SRs (20–50) were included and categorized according to patients’ treatment status into two groups: “Undergoing treatment” and “Mixed/Both phases.” Data were visualized as a bubble chart using custom HTML5/SVG code developed with AI-assisted coding (Claude, Anthropic) and Python 3.

Overall, the included reviews showed that herbal medicine has been most extensively investigated as an adjunctive therapy alongside conventional breast cancer treatments such as chemotherapy, endocrine therapy, and radiotherapy. Our findings indicate that research is predominantly concentrated in the “Undergoing treatment” phase (*n* = 27) (20–46) compared to the “Mixed/Both phase” (*n* = 4) (47–50). This trend was particularly notable in domains such as general chemotherapy-related conditions (non-symptom specific), and menopausal symptoms the majority of reviews reporting positive outcomes for herbal medicine. These results align with previous literature suggesting that complementary and integrative therapies, including herbal medicine, are primarily utilized to manage treatment-related toxicities in breast cancer patients.

Despite the increasing number of publications over the past two decades, the methodological quality of the included reviews was generally low. According to AMSTAR 2, 28 of the 31 reviews (90.3%) were rated as “Critically Low” quality and 3 (9.7%) as “Low” quality. The lowest fulfillment rates were observed in items related to protocol registration, reporting of excluded studies, funding sources of primary studies, and consideration of risk of bias. This suggests that, although the quantity of evidence on herbal medicine for breast cancer has grown rapidly, the methodological rigor and transparency of the existing reviews remain limited, which may affect the reliability of the synthesized conclusions.

Taken together, the evidence map provides a comprehensive visual landscape of current research on herbal medicine for breast cancer, highlighting both areas where evidence has accumulated and areas where substantial research gaps persist.

### Areas of major gaps in findings

This evidence map systematically maps the existing body of systematic reviews on oral herbal medicine for breast cancer, revealing both areas of research concentration and critical gaps.

First, in terms of condition domains, several clinically important areas have received markedly limited research attention. Conditions such as hot flushes, anthracycline- or chemotherapy-induced cardiotoxicity, and bone loss/osteoporosis were addressed by only one or two systematic reviews. Although some of these reviews reported positive effects, the extremely limited number of studies means the overall evidence remains sparse. Given the substantial clinical burden of these conditions in breast cancer patients—particularly the long-term cardiovascular toxicity associated with chemotherapy and the persistent impact of severe hot flushes—the lack of robust evidence in these domains represents a critical gap that warrants urgent investigation. Second, regarding outcome domains, although several reviews evaluated prognostic outcomes such as long-term survival and recurrence, none of the included reviews specifically addressed cognitive dysfunction or psychological symptoms such as anxiety and depression. Although these psychological and cognitive outcomes are central to the holistic care of breast cancer patients and survivors, the current body of systematic reviews on herbal medicine has not yet adequately covered these areas. Future research should therefore extend beyond short-term symptomatic relief to thoroughly evaluate these longer-term, patient-centered outcomes. Third, an important methodological gap was observed. Despite the relatively large volume of evidence in domains such as non-symptom specific outcomes related to general chemotherapy and menopausal symptoms, the methodological quality of the underlying reviews was consistently low. Specifically, deficiencies in protocol registration, transparent reporting of excluded studies, assessment of funding sources, and consideration of risk of bias were prevalent. This indicates that the abundance of evidence in certain domains does not necessarily equate to high-quality or trustworthy evidence, and future systematic reviews should adhere more rigorously to established methodological standards such as PRISMA (17) and AMSTAR 2 (18). Fourth, geographic and intervention-related heterogeneity represented another gap. The vast majority of included reviews originated from China, with only a few from Korea and Australia, and a wide variety of herbal formulations were evaluated without consistent standardization. This limits the generalizability of findings to other healthcare contexts and hampers cross-study comparison. Standardization of herbal medicine interventions, including formula composition, dosage, administration route, and treatment duration, and the conduct of multinational, multicenter studies are needed to enhance the external validity of future evidence. Furthermore, the term “oral herbal medicine” encompasses a heterogeneous range of interventions, including single-herb extracts, multi-herb decoctions, and standardized patent medicines. Combining all these formulations into a single category may oversimplify the evidence landscape, as different formulation types may have distinct mechanisms of action and varying levels of clinical evidence. Future evidence syntheses should consider classifying herbal medicine interventions by formulation type to enable more nuanced interpretation (51).

### Implications for practice and research

For clinical practice, the findings of this evidence map suggest that herbal medicine may be considered as an adjunctive option for managing general chemotherapy-related symptoms and endocrine therapy–induced menopausal symptoms in breast cancer patients. However, given the generally low methodological quality of the underlying evidence, clinicians should interpret these findings cautiously and take into account individual patient characteristics, potential herb–drug interactions, and the availability of conventional standard treatments.

For research, our findings highlight the need for (1) high-quality primary studies on under-investigated conditions (e.g., hot flushes and cardiotoxicity) and long-term, patient-centered outcomes; (2) standardization of herbal medicine interventions to enhance comparability across studies; (3) strict adherence to international reporting standards (e.g., PRISMA (17) and AMSTAR 2 (18)) in future systematic reviews; and (4) multinational, multicenter trials to improve the external validity and generalizability of evidence on herbal medicine in breast cancer care.

### Limitations

This evidence map has several limitations that should be acknowledged when interpreting its findings. First, only systematic reviews were included, and individual primary studies were not directly assessed. As a result, the findings reflect the secondary evidence base rather than the full body of original research on herbal medicine for breast cancer. Some clinically important primary studies that have not yet been incorporated into SRs may therefore have been overlooked (52). Second, the methodological quality of the included systematic reviews, as assessed by AMSTAR 2 (18), was generally low. Most reviews were rated as “Low” or “Critically Low” quality, with consistent deficiencies in protocol registration, transparent reporting of excluded studies, and consideration of risk of bias. Consequently, the conclusions of the included reviews—and by extension the evidence map itself–should be interpreted with caution. Third, it should be noted that AMSTAR 2 assesses the methodological quality of systematic reviews but does not evaluate the certainty of evidence for specific outcomes. Formal certainty of evidence assessment such as the GRADE (Grading of Recommendations Assessment, Development and Evaluation) approach was not applied in this evidence map, as its primary objective was to describe the distribution and volume of existing research rather than to draw conclusions about intervention effectiveness. Accordingly, the apparent concentration of SRs in certain domains particularly non-symptom specific outcomes related to general chemotherapy and menopausal symptoms should not be interpreted as indicating high certainty of evidence. The volume of available systematic reviews reflects the quantity of research activity in a given area, but not necessarily the trustworthiness or clinical applicability of the synthesized findings. Fourth, we assessed overlap among the SRs within the largest outcome cluster, “non-symptom specific (general chemotherapy)” (*n* = 13), using a citation matrix and the Corrected Covered Area (CCA). The resulting CCA was slight (0.98%), suggesting that double-counting of primary studies is unlikely to substantially inflate the apparent volume of evidence in this domain. However, this analysis was limited to the largest cluster; overlap among SRs in other outcome domains, although expected to be minimal given their distinct clinical foci, was not formally assessed. Fifth, considerable heterogeneity was observed across the included reviews in terms of populations, herbal medicine interventions (e.g., formula composition, dosage, administration route, and treatment duration), comparators, and outcome measures. This heterogeneity prevented a quantitative synthesis of effect estimates and limited direct comparison across the included reviews (19). Sixth, the included systematic reviews originated predominantly from China, with only a few from Korea and Australia, which may limit the generalizability of the findings to other healthcare contexts and patient populations outside East Asia (10, 53). Seventh, another important consideration is the heterogeneity in the quality and rigor of the journals from which the included SRs originated, while a subset of these included reviews were published in internationally indexed, peer-reviewed journals (e.g., *Cochrane Database of Systematic Reviews*, *Frontiers in Oncology*, *BMC Cancer*), a substantial proportion appeared in domestic Chinese-language journals with varying peer-review standards and limited international indexing. This heterogeneity in publication source may partly explain the consistently low AMSTAR 2 scores observed across the included reviews, as journals with less rigorous editorial and peer-review processes may be less likely to enforce adherence to established reporting standards such as PRISMA (17). Readers should therefore consider the publication source when interpreting the methodological quality and reliability of the evidence presented in this map (54, 55). Eighth, although the evidence map provides a comprehensive visual overview of the existing research landscape, it does not provide a quantitative estimate of the magnitude of effect for any specific intervention–outcome pair. The map should therefore be interpreted as a tool for identifying research gaps and trends, rather than as a substitute for quantitative meta-analysis. Finally, the literature search was limited to studies published in English, Korean, and Chinese, and reviews published in other languages may not have been captured. Furthermore, gray literature and unpublished studies were not included, which may have introduced a degree of publication bias.

## Conclusion

This evidence map provides the first comprehensive visual overview of systematic reviews on herbal medicine for breast cancer. A total of 31 SRs (20–50) were included, revealing that research has been most actively conducted on the use of herbal medicine as an adjunctive therapy during active breast cancer treatment, particularly for managing general chemotherapy-related adverse effects and endocrine therapy–induced menopausal symptoms. However, the methodological quality of the existing reviews remains uniformly low, and several clinically important outcome domains—such as cardiotoxicity, long-term survival, recurrence, and psychological symptoms—remain substantially under-investigated.

Future research should focus on expanding the evidence base in these under-researched domains, while ensuring methodological rigor and standardized intervention protocols. These findings should be interpreted in light of the predominantly low methodological quality of the included reviews and the limited geographic diversity of the evidence base. By visualizing both the clusters and gaps of current evidence, this map can serve as a useful resource for guiding future research and informing evidence-based integrative care for breast cancer patients.

## Data Availability

The original contributions presented in this study are included in the article/Supplementary material, further inquiries can be directed to the corresponding author.
