# Supplementary material for: Oral herbal medicine for breast cancer-related symptoms: an evidence map of systematic reviews
Source: Front Med (Lausanne). 2026 Aug 5;13:1903933. doi: 10.3389/fmed.2026.1903933 (PMC13485593; doi:10.3389/fmed.2026.1903933)
Supplement: Supplementary file 1 [file Table_1.docx]

**Supplementary 1. Search strategy**

**1. PubMed**

1. "Breast Neoplasms"[Mesh]

2, "breast cancer"[tiab]

3. "breast neoplasm*"[tiab]

4. "breast tum*"[tiab]

5. "breast carcinoma*"[tiab]

6. OR/ 1-5

7. "systematic review"[Publication Type]

8. "systematic review"[tiab]

9. "meta-analysis"[Publication Type]

10. "meta analysis"[tiab]

11. OR/7-10

12. 6 AND 11

**2. Cochrane library**

1. MeSH descriptor: [Breast Neoplasms] explode all trees

2."breast cancer": ti,ab,kw

3. "breast neoplasm": ti,ab,kw

4. "breast tumor": ti,ab,kw

5. "breast carcinoma": ti,ab,kw

6. OR/ 1-5

7. "systematic review" :ti,ab,kw

8. "meta-analysis" :ti,ab,kw

9. "meta analysis" :ti,ab,kw

**3. EMBASE**

1. 'breast cancer'/exp

Breast Neoplasms

2. 'breast cancer':ti,ab

3. 'breast neoplasm':ti,ab

4.
- 일반적인 유방암 코드 (Malignant Neoplasm of Breast): C

5. 'breast carcinoma':ti,ab

6 OR/ 1-5

7. 'systematic review'/de

8. 'meta analysis'/de

9. 'systematic review':ti,ab

10. 'meta-analysis':ti,ab

11. 'meta analysis':ti,ab

12. 7 OR 8 OR 9 OR 10 OR 11

**3. AMED**

**("breast cancer" OR "breast neoplasm*" OR "breast tumo?r*" )AB, TI, SU**

**AND**

**"systematic review" OR "meta-analysis"**

**4. CNKI**

1. "乳腺癌"[tiab]

2. "乳癌"[tiab]

3. "乳腺肿瘤"[tiab]

4. "乳腺恶性肿瘤"[tiab]

5. 乳房癌 [tiab]

6. OR/ 1-5

7. "系统评价"

8. "系统综述"

9. "meta分析"

10. "Meta分析"

11. "荟萃分析"

12. "系统文献回顾"

13. "systematic review"

14."meta-analysis"

15. OR/7-13

16. 6 AND 15

**5. Wanfang**

1. "乳腺癌"[tiab]

2. "乳癌"[tiab]

3. "乳腺肿瘤"[tiab]

4. "乳腺恶性肿瘤"[tiab]

5. 乳房癌 [tiab]

6. OR/ 1-5

7. "系统评价"

8. "系统综述"

9. "meta分析"

10. "Meta分析"

11. "荟萃分析"

12. "系统文献回顾"

13. "systematic review"

14."meta-analysis"

15. OR/7-13

16. 6 AND 15

**6. VIP**

1. "乳腺癌"[tiab]

2. "乳癌"[tiab]

3. "乳腺肿瘤"[tiab]

4. "乳腺恶性肿瘤"[tiab]

5. 乳房癌 [tiab]

6. OR/ 1-5

7. "系统评价"

8. "系统综述"

9. "meta分析"

10. "Meta分析"

11. "荟萃分析"

12. "系统文献回顾"

13. "systematic review"

14."meta-analysis"

15. OR/7-13

16. 6 AND 15

**7. RISS**

1. 유방암

2. 유방 종양

3. OR /1-2

4. “체계적 문헌고찰”

5. “메타분석”

6. OR/4-5

7. 3 AND 6

**8. OASIS**

1. 유방암

2. 유방 종양

3. OR /1-2

4. “체계적 문헌고찰”

5. “메타분석”

6. 고찰

7. OR/4-6

8. 3 AND 7

**9. KISS**

1. 유방암

2. 유방 종양

3. OR /1-2

4. “체계적 문헌고찰”

5. “메타분석”

6. OR/4-5

7. 3 AND 6
