# Supplementary material for: Oral herbal medicine for breast cancer-related symptoms: an evidence map of systematic reviews
Source: Front Med (Lausanne). 2026 Aug 5;13:1903933. doi: 10.3389/fmed.2026.1903933 (PMC13485593; doi:10.3389/fmed.2026.1903933)
Supplement: Supplementary file 2 [file Data_Sheet_1.zip › evidence_map_new.html]

Figure 3. Evidence Map – Oral Herbal Medicine for Breast Cancer


Figure 3. Evidence Map of Oral Herbal Medicine for Breast Cancer-Related Symptoms

Bubble size = number of systematic reviews (SRs)  | 
Bubble color = direction of effect  | 
X-axis = treatment phase and clinical condition  | 
Y-axis = predefined outcome domains (D1–D12)

Effect:

Positive

Inconclusive

No effect

Number of SRs:


1 · 5 · 9

⬇ Download as PDF
🖨 Print / Save as PDF
